# Psychometric Validation of the English and French Versions of the Posttraumatic Stress Disorder Checklist for DSM-5 (PCL-5)

**DOI:** 10.1371/journal.pone.0161645

**Published:** 2016-10-10

**Authors:** Andrea R. Ashbaugh, Stephanie Houle-Johnson, Christophe Herbert, Wissam El-Hage, Alain Brunet

**Affiliations:** 1 School of Psychology, University of Ottawa, Ottawa, Ontario, Canada; 2 Department of Psychiatry, McGill University, Montreal, Quebec, Canada; 3 Department of Psychiatry, CHRU de Tours, Université François Rabelais de Tours, Inserm U930, France; 4 Departments of Psychiatry and Neurology, McGill University, Montreal, Quebec, Canada; Universita Cattolica del Sacro Cuore Sede di Roma, ITALY

## Abstract

The purpose of this study is to assess the psychometric properties of a French version of the Posttraumatic Stress Disorder Checklist for DSM-5 (PCL-5), a self-report measure of posttraumatic stress disorder (PTSD) symptoms, and to further validate the existing English version of the measure. Undergraduate students *(n* = 838 English, *n* = 262 French) completed the PCL-5 as well as other self-report symptom measures of PTSD and depression online. Both the English and French versions PCL-5 total scores demonstrated excellent internal consistency (English: *α* = .95; French: *α* = .94), and strong convergent and divergent validity. Strong internal consistency was also observed for each of the four subscales for each version (*α’*s > .79). Test-retest reliability for the French version of the measure was also very good (*r =* .89). Confirmatory factor analysis indicated that the four-factor DSM-5 model was not a good fit of the data. The seven-factor hybrid model best fit the data in each sample, but was only marginally superior to the six-factor anhedonia model. The French version of the PCL-5 demonstrated the same psychometric qualities as both the English version of the same measure and previous versions of the PCL. Thus clinicians serving French-speaking clients now have access to this highly used screening instrument. With regards to the structural validity of the PCL-5 and of the new PTSD diagnostic structure of the DSM-5, additional research is warranted. Replication of our results in clinical samples is much needed.

## Introduction

The Posttraumatic Stress Disorder Checklist (PCL) [1] has long been a preferred measure of self-reported symptoms of posttraumatic stress disorder (PTSD). With the advent of the most recent version of the Diagnostic and Statistical Manual of Mental Disorders (DSM-5) [2], the PCL has been revised to include new symptoms and to conform to the DSM’s four-factor conceptualization of PTSD and its corresponding symptom clusters: re-experiencing, avoidance, negative alterations in cognition and mood, and increased arousal and reactivity. This shift from the previously outlined three-factor model of PTSD in DSM-IV [3] is based on a significant body of research evidence suggesting that this four-factor model best represents the structure of PTSD symptomatology [4–7]. The continuing evolution of this field highlights the notion that PTSD remains a complex spectrum type of disorder [8], making the proper measurement of it ever more important.

The PCL-5 [9] is composed of 20 items that correspond to the 20 criteria for PTSD outlined in DSM-5, and contains four subscales corresponding to the four symptom clusters mentioned above. It is a revised version of the PCL [1], which contained 17 items and three subscales corresponding to the former three symptom clusters of the DSM-IV. On the PCL-5, for each item, a score of 2 or above (range: 0 to 4, see below) is regarded as clinically relevant. In parallel with DSM-5 diagnostic guidelines, individuals can thus be accorded a tentative PTSD ‘diagnosis’ (until confirmed by a clinical interview) if they indicate scores of 2 or more on at least one re-experiencing symptom, one avoidance symptom, two symptoms of negative alterations in cognition and mood, and two arousal symptoms.

With regards to total symptom severity scores, different clinical cut-off guidelines exist for the previous 17-item PCL depending on the population and purpose of administration, ranging from 30 to 60 [10]. In general, the 17-item PCL has demonstrated superior reliability in predicting PTSD diagnosis over other measures [10].

Preliminary work on the psychometric properties of the PCL-5 has been promising. In a sample of college students, the measure demonstrated excellent internal consistency, good test-retest reliability, as well as convergent and divergent validity [11]. These results are comparable to psychometric findings for earlier versions of the measure [1,12,13], and suggest that the PCL-5 has similar psychometric rigor as previous versions. Preliminary findings regarding the appropriate cut-off scores for the PCL-5 are mixed, as reported values range from 28 to 38 [9,11]. However, no study has yet examined the psychometric properties of the PCL-5 subscales.

As mentioned above, the DSM-5 proposes a four-factor model of PTSD that is based on a large collection of research evidence. However, recent findings suggest that PTSD can also be described as having as many as six or seven factors. Research conducted by Liu and colleagues [14] suggests that PTSD is best described by six factors: intrusion, avoidance, negative affect, anhedonia, dysphoric arousal, and anxious arousal. Similarly, the seven-factor hybrid model proposed by Armour and colleagues [15] suggests that PTSD is composed of symptoms related to intrusion, avoidance, negative affect, anhedonia, dysphoric arousal, anxious arousal, and externalizing behaviour. Both models propose that positive and negative affects are best considered separately, as are dysphoric and anxious arousal. Multiple studies using the PCL-5 have demonstrated that these alternative models statistically describe PTSD better than the four-factor model proposed in DSM-5 [11,14,15].

Previous versions of the PCL were available in multiple languages, including French [16], however to our knowledge the PCL-5 is only available in English. In North America alone, there are over 20 million individuals who speak French at home [17,18]. Worldwide, French is second only to English in the number of countries that list it as an official language [19]. The most widely used version of the PCL for DSM-IV (PCL-Specific; PCL-S) has been validated and widely used in French, and demonstrates sound psychometric properties [16,20]. Similar work is lacking with regards to the development of a French version of the PCL-5.

The present study has multiple objectives. The first is to further assess the validity and reliability of the English PCL-5. The second is to evaluate the psychometric properties of a newly developed French version of the PCL-5. The internal consistency, test-retest reliability, and the convergent and discriminant validity of both English and French versions of the PCL-5 will be subject to examination. The third objective is to examine the prevalence of PTSD in our sample using the diagnostic guidelines from DSM-5 (outlined above) as well as using a cut-off score that will be identified using signal-detection analysis. The final objective of this study is to assess the structural validity of PTSD. The above analyses are run in a sample of undergraduate students at risk for PTSD.

## Methods

### Participants

Participants were undergraduate students recruited from the University of Ottawa (*n* = 1184) and McGill University (*n* = 249) in Canada. Participants at McGill University completed the study in English, and participants at the University of Ottawa had the option to complete the study in English or French. After reading the consent form online, participants implicitly consented to participate by choosing to either continue with the study or decline to proceed. This method of providing consent is considered acceptable for online studies where risk is deemed to be minimal as was the case in the present study in a non-clinical sample [21]. All participants received course credit in exchange for participation. All study and consent procedures were approved by University of Ottawa Health Sciences Ethics Board and the McGill Faculty of Medicine Institutional Review Board.

### Procedure

After providing informed consent, participants completed a set of online questionnaires (see below). After completing the questionnaires (time 1), participants were invited to complete the questionnaires a second time to assess test-retest reliability (time 2). Forty-five participants in the English sample completed the questionnaires a second time, however, results are not reported as the rate of participation (5%) in the retest portion of the study was considered to be too low (<80% power to detect medium ICC at alpha = .05). Though rates of participation in the French sample were also low (16%) results are reported as no previous study has presented psychometric properties of the French version of the PCL-5, and statistical power analysis demonstrated adequate power to run the ICC analyses in this group.

### Measures

#### Life Events Checklist (LEC) [22]

The LEC is a 17-item checklist assessing exposure to potentially traumatic events [22]. Respondents are asked to indicate whether they have experienced, witnessed, or learned about 17 different traumatic events, or any other particularly distressing experiences not encompassed by the other 17 items. Participants were asked to identify an “index event” (the event that caused them the most distress as of this day) to refer to for the remainder of the study. The LEC has demonstrated adequate stability in samples of both university students and combat veterans [22], and has demonstrated strong convergent validity [22]. The French version of the LEC was translated by a French-English bilingual expert on traumatic stress (A.B.).

#### Posttraumatic Stress Disorder Checklist– 5 (PCL-5) [9]

The PCL-5 is a 20-item self-report inventory assessing the severity of PTSD symptoms for the past month, as per the DSM-5. The PCL-5 has 4 subscales, corresponding to each of the symptom clusters in the DSM-5. Respondents rated how much a problem described in the item statement bothered them over the past month on a 5-point scale from 0 (not at all) to 4 (extremely). Scores on the PCL-5 range from 0–80. The French version of the PCL-5 was translated by a French-English bilingual researcher (A.B.) and back translated by bilingual experts on traumatic stress from Canada and France (A.A. and W.E.H.). The translated measure was presented to focus groups of patients in Canada and France as part of a cultural validation process. Minor edits were subsequently made. The French version of the PCL-5 is available upon request.

#### Impact of Event Scale–Revised (IES-R) [23]

The IES-R is a 22 item self-report measure of PTSD symptom severity with three subscales assessing intrusions, avoidance, and hyperarousal in the past 7 days. The IES-R has demonstrated consistent test-retest reliability and excellent internal consistency, as well as both convergent and divergent validity [23]. The French version of the IES-R has also demonstrated excellent psychometric properties [24]. In the current study the internal consistency in the English and French samples respectively was .96 and .95 for the total IES-R, .92 and .91 for the intrusion subscale, .91 and .88 for the avoidance subscale, and .89 and .90 for the arousal subscale. The IES-R was used to assess convergent validity of the PCL-5.

#### Center for Epidemiological Studies–Depression Scale (CES-D) [25]

The CES-D is a 20-item self-report measure assessing current depressive symptoms [26]. The empirically validated French version of the CES-D was used for the French sample [27]. Participants were asked to rate how often they experience each symptom on a 4-point scale ranging from “rarely or none of the time” (less than one day) to “all of the time” (5–7 days) [26]. The CES-D has demonstrated good internal consistency, test-retest reliability, and acceptable convergent and divergent validity [25]. The internal consistency of the CES-D in our study was .89 and .92 for the English and French samples, respectively. The CES-D was used to assess divergent validity of the PCL-5.

#### Statistical Analyses

Validity and reliability analyses were conducted using SPSS version 22.0 [28], and factor analyses were conducted using SPSS AMOS version 23.0 [29]. Alpha was calculated for the total PCL-5 and its subscales to assess internal consistency. In the French sample, intraclass correlation coefficients were calculated using scores from time 1 and time 2 to determine test-retest reliability. Convergent validity was assessed via correlations between the PCL-5 and the IES-R, and between the PCL-5 subscales and their corresponding IES-R subscales. Using the Fisher *r*-to-*z* transformation we compared the magnitude of the correlation between the PCL-5 and the IES-R to that observed between the PCL-5 and the CES-D to assess divergent validity.

Signal-detection analyses were conducted using the DSM-5 diagnostic guidelines applied to the PCL-5 to dichotomize participants into ‘Probable PTSD’ and ‘Non-PTSD’ groups, as suggested by Weathers et al. [2,9]. Thus participants with scores 2 or above on at least one re-experiencing symptom, one avoidance symptom, two symptoms of negative alterations in cognition and mood, and two arousal symptoms were classified as having probable PTSD. Using the results of a previous study as a starting point [11], PCL-5 scores were examined to determine which best predicted the prevalence of probable PTSD as per this grouping. The score that yielded a prevalence proportion that most closely reached that determined by the DSM-5 guidelines (without exceeding it), and with the highest specificity, sensitivity and efficiency ratings, was selected.

Three structural models of PTSD were tested using confirmatory factor analysis (CFA). The first tested the DSM-5 four-factor model of PTSD, using the four PCL-5 subscales. The second tested the six-factor anhedonia model [14], and the third tested the seven-factor hybrid model of PTSD [15]. In each case, maximum likelihood estimation procedure was applied, and factor variance for each latent variable was set to 1. Because latent variables were theoretically expected to correlate and to ensure the models were properly identified, latent variables were allowed to correlate with one another. Goodness-of-fit indices were interpreted according to guidelines by Hu and Bentler [30], thus adequate model fit was determined based on cut-offs of ≥ .95 for the comparative fit index (CFI), ≤ .06 for the root mean square error of approximation (RMSEA) and ≤ .08 for the standardized root mean square (SRMR). In order to compare models, chi-square difference tests and the Akaike information criterion (AIC) were examined. Regarding the AIC, the lowest value of those produced by each model indicates better comparative fit. An analysis of measurement invariance was also performed in order to test the potential differences in fit between the English and French versions of the measure. Less than 2% of the PCL-5, IES-R and CES-D values were missing, thus a single imputation was performed.

## Results

**English PCL-5.** Only responses from participants who reported a DSM-5 traumatic event were analysed. Thus, of the 1098 English participants, 72 participants were excluded because their index event did not meet DSM-5 criterion A and another 95 participants were excluded because they endorsed does not apply” on the LEC. An additional 93 participants were excluded for the following reasons: completed less than 50% of the PCL-5 (*n* = 70); declined to submit data for analysis (*n* = 9); participant had more than one of these issues with their data (*n* = 14). Thus, 838 participants were retained for analysis. The included sample had a significantly higher proportion of females than the excluded sample (*x*^*2*^ [1] *=* 7.73, *p* < .05), however no additional differences were observed.

Table 1 presents the characteristics of the English sample, and Table 2 presents the frequency of endorsed LEC events. Means, standard deviations, minimum and maximum values for all measures are presented in Table 3.

**Table 1:** Sociodemographic Characteristics of the Sample.

			English sample (*n* = 838)			French sample (*n* = 262)		
Characteristics			%	*n*	Missing (*n*)	%	*n*	Missing (*n*)
Sex					11			2
	Female	76.7		643		80.2	210	
	Male	22.0		184		19.1	50	
Age (*M*, *SD*)		20.0		4.1	3	20.0	2.8	0
Race					27			10
	Caucasian	57.8		484		59.9	157	
	Black	8.2		69		18.3	48	
	Hispanic	1.6		13		1.5	4	
	Asian	16.0		134		3.4	9	
	Native American	1.7		14		0.8	2	
	European	3.3		28		3.1	8	
	Other	8.2		69		9.2	24	
Marital Status					15			4
	Married	3.0		25		3.1	8	
	Living Together	5.1		43		6.9	18	
	Separated/Divorced	0.5		4			0	
	Widow	0.1		1		0.4	1	
	Single	89.5		750		88.2	231	
Education Level								14
	Some college/AA degree/Technical school training	53.3		447		63.4	166	
	College Graduate (Bachelor’s)	39.1		328		30.2	79	
	Graduate school degree: Master’s or Doctorate degree	2.9		24		1.1	3	
Annual Household Income					37			10
	Less than $20,000	44.5		373		72.1	189	
	$20,000–40,000	9.5		80		8.0	21	
	$40,001–60,000	11.7		98		5.7	15	
	$60,001–80,000	10.6		89		3.1	8	
	More than $80,000	19.2		161		7.3	19	
Past PTSD^a^ diagnosis					15			6
	No	95.7		802		93.9	246	
	Yes	2.5		21		3.8	10	
Past PTSD treatment					10			5
	Never	96.3		807		93.5	245	
	Yes, in the past	1.9		16		2.7	7	
	Yes, currently	0.6		5		1.9	5	
First Language					9			3
	English	65.6		550		11.8	31	
	French	17.4		146		78.2	205	
	Other	15.9		133		8.8	23	
Fluency in Language of Survey^1^					9			3
	Native speaker, totally fluent (100%)	78.8		660		75.2	197	
	Understand almost everything (90+%)	13.2		111		16.8	44	
	Understand a lot (80–90%)	4.5		38		3.4	9	
	Understand about 70–80%	1.6		13		1.9	5	
	Understand about 50–70%	0.8		7		1.5	4	

^a^Posttraumatic stress disorder.

^1^Note: Additional analyses were conducted using only participants with >90% fluency in the survey language. Results did not differ between the two analyses, thus the full sample was retained.

**Table 2:** Proportion of potentially traumatic events endorsed as index event.

	English sample (*n =* 838)		French sample (*n =* 262)	
Event	%	*n*	%	*n*
Sudden and unexpected death of someone close to you	18.9	158	19.1	50
Transportation accident	17.1	143	21.3	56
Life-threatening illness or injury	9.7	81	11.1	29
Sexual assault	6.1	51	8.4	22
Natural disaster	5.8	49	7.6	20
Physical assault	5.7	48	7.3	19
Other unwanted or uncomfortable sexual experience	5.7	48	2.3	6
Physical or sexual abuse during childhood	5.5	46	1.5	4
Other traumatic event	5.1	43	3.1	8
Sudden violent death	5.0	42	5.3	14
Serious accident at work, home or during recreational activity	4.1	34	1.9	5
Fire or explosion	3.9	33	3.8	10
Serious injury, harm or death you caused to someone close to you	2.6	22	0.0	0
Severe human suffering	2.3	19	2.3	6
Combat or exposure to a war-zone	1.2	10	2.3	6
Assault with a weapon	0.6	5	2.7	7
Exposure to a toxic substance	0.5	4	0.0	0
Captivity	0.2	2	0.0	0
Endorsed 1 traumatic event	2.1	18	3.1	8
Endorsed 2 traumatic events	2.9	24	4.2	11
Endorsed 3 traumatic events	5.5	46	10.7	28
Endorsed 4 or more traumatic events	89.5	750	82.0	215

**Table 3:** Normative Data for the PCL-5, IES-R, and CES-D.

	English sample (*n* = 838)				French sample (*n* = 262)			
Scale	*M*	*SD*	Min.	Max.	*M*	*SD*	Min.	Max.
PCL-5^a^								
Intrusion	5.6	4.9	0	20	5.8	4.5	0	18
Avoidance	2.7	2.4	0	8	2.7	2.5	0	8
Cogn.^b^/Mood	7.1	6.9	0	28	6.7	6.5	0	26
Arousal	5.5	5.3	0	24	5.2	5.3	0	24
Total	20.9	17.7	0	80	20.4	16.7	0	68
IES-R^c^								
Intrusion	7.4	7.4	0	31	9.4	7.9	0	29
Avoidance	8.7	8.1	0	32	9.7	7.8	0	32
Arousal	4.5	5.3	0	24	5.4	6.0	0	24
Total	20.6	19.4	0	88	24.5	19.9	0	81
CES-D^d^								
Total	16.5	11.4	0	54	18.3	11.0	3	53

^a^Posttraumatic Stress Disorder Checklist-5

^b^Cognition

^c^Impact of Events Scale-Revised

^d^Center for Epidemiological Studies Depression Scale

**Internal Consistency.** As seen in Table 4, the PCL-5 demonstrated excellent internal consistency. Cronbach’s alphas for each of the subscale scores were also very high.

**Table 4:** Reliability coefficients for the PCL-5 (English and French versions).

	English sample	French sample
Scale	Cronbach’s alpha (*n* = 838)	Cronbach’s alpha (*n* = 262)
PCL-5^a^^b^		
Intrusion	.88	.83
Avoidance	.81	.79
Cogn^c^/Mood	.90	.87
Arousal	.85	.87
Total score	.95	.94

^a^Intraclass correlation coefficient

^b^Posttraumatic Stress Disorder Checklist-5

^c^Cognition

**Convergent and Divergent Validity.** The correlation between the PCL-5 and the IES-R yielded a significant, positive correlation (*r* = .82, *p* < .001) suggesting strong convergent validity. Regarding the corresponding PCL-5 and IES-R subscales, a positive, statistically significant correlation was observed in each case (intrusion: *r* = .76; avoidance: *r =* .68; arousal: *r =* .81, all *p* < .001).

The correlation between the PCL-5 and the CES-D yielded a coefficient of *r* = .64, (*p* < .001), and was significantly lower than that observed between the PCL-5 and IES-R, (*z* = 8.15, *p* < .01), supporting the measure’s divergent validity.

**Signal Detection Analysis.** The prevalence of participants with provisional PTSD as assessed by applying the DSM-5’s diagnostic guidelines to the PCL-5 [9] was 26.8%. Signal-detection analysis revealed that a PCL-5 cut-off score of 31 best predicted this PTSD diagnostic grouping based on the DSM-5, yielding a prevalence of 26.3% with a specificity of .95, sensitivity of .85, and an efficiency of .95.

**Factor Structure.** For the four-factor model, only the SRMR value indicated adequate fit [30] (see Table 5). For both the six and seven factor models, the values for all fit indices reached the appropriate cut-off levels (Table 5). The six-factor model had significantly better fit than the four-factor model, (*x*^*2*^ [9] *=* 450.73, *p* < .05), and the seven-factor model demonstrated superior fit to the six-factor model (*x*^*2*^ [6] *=* 49.59, *p* < .05). In addition, the AIC value for the seven-factor hybrid model was the lowest (Table 5). Together, these results suggest that the seven-factor hybrid model best fit the data. Standardized parameter estimates and factor correlations for each of these models can be found in Tables 6 and 7, respectively.

**Table 5:** Results of the confirmatory factor analyses: Four-factor DSM-5 model, six-factor anhedonia model and seven-factor hybrid model and English vs. French measurement invariance.

	English sample (*n* = 838)			French sample (*n* = 262)			English vs. French Measurement Invariance		
Fit criterion	DSM-5 model	6-factor anhedonia model	7-factor hybrid model	DSM-5 model	6-factor anhedonia model	7-factor hybrid model	DSM-5 model	6-factor anhedonia model	7-factor hybrid model
*x*^2^^a^	1139.79	689.60	640.06	506.60	400.85	386.24	1646.70	1090.82	1026.68
*Df*	164	155	149	164	155	149	328	310	298
CFI^b^	.91	.95	.96	.89	.92	.92	.91	.94	.95
RMSEA^c^	.08	.06	.06	.09	.08	.08	.06	.05	.05
RMSEA CI^d^ (95)	.08-.09	.06-.07	.06-.07	.08-.10	.07-.09	.07-.09	.06-.06	.04-.05	.04-.05
SRMR^e^	.05	.04	.03	.05	.05	.05	.05	.04	.05
AIC^f^	1231.79	799.60	762.06	598.60	510.85	508.24	1830.69	1310.82	1270.67

^a^All p values < .001

^b^Comparative fit index (cut-off ≥ .95)

^c^Root mean square error of approximation (cut-off ≤ .06)

^d^Confidence interval (95%)

^e^Standardized root mean square (cut-off guideline ≤ .08)

^f^Akaike information criterion (lowest observed value indicates better model fit)

**Table 6:** Standardized parameter estimates and associated factor items for confirmatory factor analysis models–English sample.

	DSM-5 model		Six-factor anhedonia model		Seven-factor hybrid model	
PCL-5 item	Factor	Factor	Estimate	Factor	Factor	Estimate
1. Disturbing memories of experience	R^a^	.82	R	.82	R	.82
2. Disturbing dreams of experience	R	.73	R	.72	R	.72
3. Suddenly feeling or acting as if the stressful experience were actually happening again	R	.76	R	.76	R	.76
4. Upset when reminded of stressful experience	R	.77	R	.77	R	.77
5. Physical reactions to reminders of the experience	R	.75	R	.75	R	.75
6. Avoiding memories, thoughts or feelings related to experience	Av^b^	.85	Av	.85	Av	.85
7. Avoiding external reminders of the stressful experience	Av	.81	Av	.81	Av	.81
8. Trouble remembering experience	NACM^c^	.46	NA^d^	.47	NA	.47
9. Negative beliefs of self, other people and the world	NACM	.78	NA	.80	NA	.80
10. Blaming self or others for experience	NACM	.74	NA	.81	NA	.81
11. Having strong negative feelings such as fear, horror, anger, guilt, or shame	NACM	.79	NA	.85	NA	.85
12. Loss of interest in activities	NACM	.80	AH^e^	.84	AH	.84
13. Feeling distant or cut-off from other people	NACM	.82	AH	.87	AH	.87
14. Trouble experiencing positive feelings	NACM	.84	AH	.86	AH	.86
15. Irritability, angry outbursts, or acting aggressively	Ar^f^	.76	DYS^g^	.77	EX^h^	.79
16. Taking too many risks or doing things that could cause you harm	Ar	.69	DYS	.69	EX	.71
17. Being “superalert” or watchful or on guard	Ar	.54	ANX^i^	.69	ANX	.69
18. Feeling jumpy or easily startled	Ar	66	ANX	.83	ANX	.83
19. Having difficulty concentrating	Ar	.80	DYS	.25	DYS	.83
20. Trouble falling or staying asleep	Ar	.71	DYS	.70	DYS	.73

^a^Re-experiencing/intrusion

^b^Avoidance

^c^Negative alterations in cognition and mood

^d^Negative affect

^e^Anhedonia

^f^Increased arousal and reactivity

^g^Dysphoric arousal

^h^Externalizing behaviour

^i^Anxious arousal.

**Table 7:** Correlations among latent variables for confirmatory factor analysis models.

	English (*n* = 838)			French (*n* = 262)		
	Factors Correlated		Estimate	Factors Correlated		Estimate
**4-factor DSM-5 model**						
	R	Av	.842	R	Av	.864
	R	Ar	.891	R	Ar	.895
	R	NACM	.815	R	NACM	.805
	Av	Ar	.778	Av	Ar	.671
	Av	NACM	.822	Av	NACM	.703
	Ar	NACM	.920	Ar	NACM	.903
**6-factor anhedonia model**						
	R	Av	.843	R	Av	.866
	R	NA	.819	R	NA	.856
	R	AH	.740	R	AH	.698
	R	DYS	.879	R	DYS	.883
	R	ANX	.728	R	ANX	.809
	Av	NA	.824	Av	NA	.759
	Av	AH	.749	Av	AH	.592
	Av	DYS	.769	Av	DYS	.657
	Av	ANX	.634	Av	ANX	.618
	NA	AH	.834	NA	AH	.850
	NA	DYS	.828	NA	DYS	.867
	NA	ANX	.696	NA	ANX	.766
	AH	DYS	.926	AH	DYS	.888
	AH	ANX	.616	AH	ANX	.677
	DYS	ANX	.759	DYS	ANX	.857
**7-factor hybrid model**						
	R	Av	.843	R	Av	.866
	R	NA	.819	R	NA	.855
	R	AH	.740	R	AH	.694
	R	DYS	.879	R	DYS	.906
	R	EX	.823	R	EX	.847
	R	ANX	.728	R	ANX	.807
	Av	NA	.824	Av	NA	.759
	Av	AH	.749	Av	AH	.587
	Av	DYS	.749	Av	DYS	.666
	Av	EX	.744	Av	EX	.640
	Av	ANX	.633	Av	ANX	.616
	NA	AH	.834	NA	AH	.846
	NA	DYS	.786	NA	DYS	.838
	NA	EX	.826	NA	EX	.896
	NA	ANX	.695	NA	ANX	.763
	AH	DYS	.855	AH	DYS	.827
	AH	EX	.950	AH	EX	.945
	AH	ANX	.616	AH	ANX	.672
	DYS	EX	.917	DYS	EX	.994
	DYS	ANX	.761	DYS	ANX	.877
	EX	ANX	.709	EX	ANX	.828

Note: Factor abbreviations are as follows: R: re-experiencing; Av: avoidance; NACM: negative alterations in cognition and mood; NA: negative affect; AH: anhedonia; Ar: increased arousal and reactivity; DYS: dysphoric arousal; EX: externalizing behaviour; ANX: anxious arousal.

### French PCL-5

As with the English sample, only participants who reported an index event that corresponded to DSM-5 criterion A were included. Thus of the 335 French speaking participants, 15 were excluded because trauma specified did not meet DSM-5 criterion A and 37 were excluded because they either indicated “does not apply” on the LEC or did not indicate an index event. An additional 36 French-speaking participants were excluded because they completed less than 50% of the PCL-5. Thus, 262 trauma-exposed participants were included in the final sample. These participants were not statistically different from the excluded participants on any of the descriptive variables (all *ps* > .05). Of these participants, 42 provided complete test-retest data. While this response rate is relatively low (16%), post-hoc calculations determined that this re-test sample size was adequate to achieve 99% power in detecting the observed ICC [31]. Participants who completed the study at time 2 were slightly older than those who did not complete the study at re-test, *t*(260) = 2.38, *p* < .05, but were not statistically different on any other sociodemographic variable. No differences were observed with regards to initial PCL-5, IES-R or CES-D scores between the two groups.

Table 1 presents the characteristics of the French sample, and Table 2 presents the frequency of endorsed LEC events. Means, standard deviations, minimum and maximum values for all measures are presented in Table 3.

#### Internal Consistency

Table 4 demonstrates the internal consistency for the total PCL-5 and the subscales, which all yielded sufficiently high coefficients.

#### Test-retest

The average number of days between time 1 and time 2 was 20.95 days (*SD* = 22.11, range: 5 to 144 days). The total scale demonstrated very good test-retest reliability (ICC = .89, 95% CI = .78-.94, *p* < .001), as did the intrusion (ICC = .80, 95% CI = .63-.89, *p* < .001), negative alterations in cognition and mood (ICC = .92, 95% CI = .85-.96, *p* < .001) and arousal (ICC = .78, 95% CI = .60–.88, *p* < .001) subscales. The intraclass correlation coefficient for the avoidance subscale did not meet standards of acceptable reliability (ICC = .66, 95% CI = .37–.82, *p* = .009) [32].

#### Convergent and Divergent Validity

The correlation between the PCL-5 and IES-R yielded a significant result (*r* = .80, *p* < .001). Here again, a strong positive correlation was observed between the corresponding PCL-5 and IES-R subscales (intrusion: *r* = .71; avoidance: *r =* .65; arousal: *r =* .78, all *p* < .001).

The correlation between the PCL-5 and the CES-D was .62 (*p* < .001), and was significantly lower than the correlation observed between the PCL-5 and the IES-R (*z* = 4.25, *p* < .001), supporting the divergent validity of the PCL-5.

#### Signal Detection Analysis

Using DSM-5 diagnostic guidelines [9], the prevalence of PCL-5 provisional PTSD was 24.0%. Signal-detection analysis determined that a score of 32 on the PCL-5 yielded similar prevalence of 'probable PTSD' (23.7%), with a specificity of .95, a sensitivity of .83 and an efficiency of .92.

#### Factor Structure

For all three CFA models, only the SRMR value attained the acceptable cut-off value (see Table 5). However, fit of the six-factor model yielded significantly better fit than the four-factor model (*x*^*2*^ [9] *=* 106.16, *p* < .05), with the seven-factor model yielding the best fit for the data (six-factor vs. seven-factor model: *x*^*2*^ [6] *=* 14.66, *p* < .05). Further, as in the English sample, the lowest AIC value observed was for the seven-factor hybrid model. Standardized parameter estimates and factor correlations for each model are shown in Tables 8 and 7, respectively. Results of the measurement invariance analyses demonstrated the same pattern of results as above, with the seven-factor hybrid model yielding the best fit when both samples were included in the CFAs, demonstrating configural invariance (see Table 5).

**Table 8:** Standardized parameter estimates and associated factor items for confirmatory factor analyses models–French sample.

	DSM-5 model		Six-factor anhedonia model		Seven-factor hybrid model	
PCL-5 item	Factor	Estimate	Factor	Estimate	Factor	Estimate
1. Disturbing memories of experience	R^a^	.76	R	.76	R	.82
2. Disturbing dreams of experience	R	.65	R	.65	R	.72
3. Suddenly feeling or acting as if the stressful experience were actually happening again	R	.67	R	.66	R	.76
4. Upset when reminded of stressful experience	R	.71	R	.72	R	.77
5. Physical reactions to reminders of the experience	R	.74	R	.74	R	.75
6. Avoiding memories, thoughts or feelings related to experience	Av^b^	.82	Av	.82	Av	.85
7. Avoiding external reminders of the stressful experience	Av	.80	Av	.80	Av	.81
8. Trouble remembering experience	NACM^c^	.43	NA^d^	.47	NA	.47
9. Negative beliefs of self, other people and the world	NACM	.72	NA	.73	NA	.80
10. Blaming self or others for experience	NACM	.67	NA	.69	NA	.81
11. Having strong negative feelings such as fear, horror, anger, guilt, or shame?	NACM	.78	NA	.83	NA	.85
12. Loss of interest in activities	NACM	.77	AH^e^	.78	AH	.84
13. Feeling distant or cut-off from other people	NACM	.86	AH	.93	AH	.87
14. Trouble experiencing positive feelings	NACM	.70	AH	.73	AH	.86
15. Irritability, angry outbursts, or acting aggressively	Ar^f^	.79	DYS^g^	.80	EX^h^	.79
16. Taking too many risks or doing things that could cause you harm	Ar	.61	DYS	.62	EX	.71
17. Being “superalert” or watchful or on guard	Ar	.68	ANX^i^	93	ANX	.69
18. Feeling jumpy or easily startled	Ar	.80	ANX	.90	ANX	.83
19. Having difficulty concentrating	Ar	.82	DYS	.81	DYS	.83
20. Trouble falling or staying asleep	Ar	.65	DYS	.66	DYS	.73

^a^Re-experiencing/intrusion

^b^Avoidance

^c^Negative alterations in cognition and mood

^d^Negative affect

^e^Anhedonia

^f^Increased arousal and reactivity

^g^Dysphoric arousal

^h^Externalizing behaviour

^i^Anxious arousal.

## Discussion

The current study examined the psychometric properties of the English version of the PCL-5 and a newly developed French version in a sample of trauma-exposed undergraduate students. Both versions of the PCL-5 proved to be psychometrically sound, as each demonstrated excellent internal consistency, and strong convergent and divergent validity. Internal consistencies for the PCL-5’s subscales were also very high for both versions of the measure. Further, test-retest reliability for the newly developed French version of the measure was very good.

This study is the first to present a French-language version of the PCL-5. The practical implications of this are widespread, as important research has been done using the French version of the PCL-S [16], which has been cited 135 times according to Google Scholar. This updated version is now available for use among French speaking populations.

While the study sample did include both native and non-native speakers of both English and French, the results did not change when excluding those with <90% fluency in the survey language. Given that Canada is a culturally diverse population, with over 20% of the population having a mother language other than English or French [33], we believe that the results of the full sample speak to the generalizability of the PCL-5 in both languages, which can likely be applied without issue in other diverse English and French speaking countries.

Overall, the fit indices observed for each of the CFA models in the French sample were similar to those observed in the English sample, but did not meet the predetermined criteria outlined for this study. It is worth noting, however, that the same pattern of model fit was observed when comparing the three models tested in both samples, with the seven-factor model yielding the best fit. This pattern is further supported by analyses of measurement invariance, which suggest that the two samples fit the data similarly across languages. Further, certain commonly cited sources consider a RMSEA value of ≤ .08 and a CFI of ≥ .90 to indicate acceptable fit [34–36]. Indeed, it was by these specifications that Blevins and colleagues [11] evaluated these same models. Thus, by these standards both the six- and seven-factor models achieved acceptable fit in the French sample. Another explanation for the lower observed fit indices in the French sample may be that the sample was slightly smaller than is recommended to run factor analyses [37]. This is the first study to examine the factor structure of PTSD using the PCL-5 in a French sample, thus replication of these results in larger samples of French-speakers is warranted. Furthermore, the factor structure was examined in a population of trauma-exposed individuals rather than individuals with PTSD. More research into the structural validity of the PCL-5 among clinical samples in both English and French is also much needed.

The prevalence of probable PTSD was relatively high in this sample of university students compared to that observed in the Blevins et al. study [11] This is likely due to the specificity with which the presence of trauma was assessed in our study. Here, participants were asked to refer to the most distressing experience endorsed on the LEC when completing the questionnaires. In contrast, Blevins and colleagues [11] simply asked students to report whether they had experienced “a very stressful life event.”

It is also notable that a very large proportion of participants endorsed having experienced a traumatic event in the current study, though it was not a requirement for participation. Approximately 85% of the English sample and 83% of the French-speaking sample reported having experienced a traumatic event. However, a large proportion of reported events are relatively common events (e.g., transportation accident, sudden unexpected death of someone close) and fewer participants reported arguably more severe traumatic events, such as sexual or physical assault. Furthermore, previous studies have found between 40% to 85% of undergraduate students report having experienced a traumatic event [38–40]. Thus current findings seem to support previous research suggesting that traumatic events are relatively common phenomena in at least undergraduate samples. Further, given that the sample represents one in which the risk of PTSD is high, the psychometric findings presented here will likely generalize well to clinical samples.

As suggested by Weathers et al. [9] the current study applied the DSM-5 diagnostic guidelines to the PCL-5 to determine prevalence of PTSD and then to determine a PCL-5 cut-off score. A PCL-5 score of 31 in the English sample and 32 in the French sample was deemed to have the greatest likelihood of correctly categorizing a participant as having or not having probable PTSD as per the DSM-5 guidelines. In contrast to the procedure applied in the Blevins et al. [11] study, the criteria applied in the signal-detection analyses reflect the DSM-5 model of PTSD rather than the DSM-IV-TR conceptualization of the disorder [3]. Thus, the cut-off values identified here may be more clinically useful for those using the DSM-5 than the score proposed by other researchers. However, no study has yet examined cut-off scores using strict clinical guidelines. Thus, to gain a more accurate indication of the PCL-5 cut-off scores that best predict actual PTSD diagnosis, future research should examine potential PCL-5 cut-off scores using clinician-administered measures designed to adhere more strictly to the DSM-5 symptomatology of PTSD, such as the Clinician-Administered PTSD Scale for DSM-5, [41].

We found that a seven-factor hybrid model of PTSD in which negative and positive affect, anxious and dysphoric arousal and externalizing behaviour are separate factors, best fit the data in both the English and French samples. Statistically the inclusion of this many factors is said to be problematic by some experts, especially when multiple factors have only two items per factor, as composite scores for these factors are likely unreliable [32]. Indeed, the low test-retest coefficient for the avoidance subscale of the PCL-5 can likely be explained by the fact that it contains only two items. However, many previously proposed models of PTSD have included two-item factors, including DSM-5 four-factor model. Theoretically, allowing latent factors to covary allows for the model to be properly identified, making the interpretation of these models rather straightforward [42]. Further, the strength of the seven-factor model over others has been demonstrated in several studies already [11,14,15], adding to its credibility as a potential theoretical model of PTSD. While it is not within the scope of this study to discuss the potential reconceptualization of the structure of PTSD, it is clear that further psychometric work is needed to assess the predictive validity and clinical utility of alternative, more comprehensive theories of PTSD. At the very least it can be said that the diversity of constructs assessed by both the negative alterations in cognition and mood and the increased arousal and reactivity dimensions of PTSD may indeed provide clinicians and researchers with additional information regarding the symptomatology, diagnosis and treatment of PTSD see [14,15,43,44] for additional information. At this point, we recommend using the DSM-5 guidelines described above, or a cut-off score of 31 to determine provisional PTSD requiring further clinical attention, though again we emphasize the need for our findings concerning the factor structure and recommended cut-offs to be replicated in a sample of individuals diagnosed with PTSD.

## Conclusion

This study is the first to present a French-language version of the PCL-5, which demonstrated psychometric properties akin to those observed for both the original English-language version of the 17-item PCL and the English PCL-5. Overall, the total score of the PCL-5 in both the French and English demonstrated excellent reliability, as well as convergent and divergent validity. Using CFA, our data demonstrated better fit with the six-factor anhedonia model and the seven-factor hybrid model compared to the four-factor DSM-5 model, with the seven-factor model slightly surpassing the six-factor model in fit. Future research should continue to examine the differentiation of the DSM-5 symptom groups for the cognition and mood and the increased arousal and reactivity dimensions of the disorder. Replication of these results in clinical samples is much needed, as no research has yet assessed the validity of the PCL-5 in these populations.
